# Expanding the *MRPS34* Genotype–Phenotype Correlation: Two Novel Cases and a Cohort Review

**DOI:** 10.1002/jmd2.70089

**Published:** 2026-06-08

**Authors:** Alberte Aspaas Lundquist, Sumit Parikh, Thomas van Overeem Hansen, Flemming Wibrand, Elsebet Østergaard

**Affiliations:** ^1^ Department of Clinical Genetics, Rigshospitalet Copenhagen University Hospital Copenhagen Denmark; ^2^ Department of Paediatrics and Adolescent Medicine, Rigshospitalet Copenhagen University Hospital Copenhagen Denmark; ^3^ Department of Congenital Disorders Statens Serum Institut Copenhagen Denmark; ^4^ Department of Clinical Medicine University of Copenhagen Copenhagen Denmark; ^5^ Department of Child Neurology Cleveland Clinic Children's Hospital Cleveland Ohio USA

**Keywords:** combined oxidative phosphorylation deficiency 32, genotype–phenotype correlation, hypomorphic splice variant, leigh syndrome spectrum, mitochondrial disease, MRPS34

## Abstract

*MRPS34* encodes a mitoribosomal protein essential for mitochondrial translation. Biallelic pathogenic variants in *MRPS34* cause Combined Oxidative Phosphorylation Deficiency 32 (COXPD32), a rare mitochondrial disorder within the Leigh syndrome spectrum (LSS), ranging from fatal in infancy to adult survival. The objective is to describe two new individuals with *MRPS34*‐related disease and expand the clinical, genetic, and phenotypic spectrum of COXPD32. Clinical, radiological, biochemical, and molecular evaluations were conducted in two individuals with Leigh Syndrome (LS). Exome and genome sequencing identified presumed biallelic *MRPS34* variants. A systematic review of all previously reported cases was performed to assess possible genotype–phenotype correlations (*n* = 11). Individual 1, who died in infancy with LS, was presumed compound heterozygous for a novel splice‐site variant (c.364 + 2 T>C, p.(?)) and a nonsense variant (c.94C>T, p.(Gln32*)). Individual 2 survived into mid childhood and was homozygous for the hypomorphic variant c.322‐10G>A, p.(?). Among 11 individuals, key features included developmental delay (100%), lactic acidosis (91%), brainstem lesions (91%), and metabolic acidosis (83%). Homozygosity for c.322‐10G>A, p.(?) correlated with longer survival. *MRPS34*‐related disease presents with multisystemic features and genotype‐dependent severity. Accurate genetic diagnosis is essential for prognosis and therapeutic strategies.

## Key Points

- *MRPS34*‐related disease (COXPD32) is associated with developmental delay, brainstem involvement, lactic acidosis, and respiratory chain deficiency within the Leigh syndrome spectrum.
- We report two additional individuals, including a novel splice‐site variant, expanding the genetic and phenotypic spectrum of COXPD32.
- The hypomorphic c.322‐10G>A variant is associated with longer survival, although neurodevelopmental impairment remains.

## 1 Introduction

Leigh syndrome (LS; MIM: 256000), or subacute necrotizing encephalomyelopathy, is a rare progressive neurodegenerative disorder caused by defects in mitochondrial energy metabolism and characterized by lesions in the basal ganglia and brainstem [1, 2]. Leigh‐like syndrome (LLS) describes disorders with similar clinical and radiological features that do not meet diagnostic criteria for LS; together, LS and LLS comprise the Leigh syndrome spectrum (LSS).

Mitochondria generate energy through oxidative phosphorylation (OXPHOS), which depends on components encoded by nuclear DNA (nDNA) and mitochondrial DNA (mtDNA). Variants in over 75 OXPHOS‐related genes have been linked to LS and related disorders [1, 3]. Mitochondrial protein synthesis is performed by mitoribosomes, composed of a small (28S) subunit, which decodes messenger RNA, and a large (39S) subunit for peptide bond formation [4, 5, 6]. These subunits produce 13 mtDNA‐encoded proteins essential for OXPHOS [5, 7]. The small (28S) subunit consists of 12S ribosomal RNA (rRNA) and around 30 mitochondrial ribosomal small subunit proteins (MRPSs), whereas the large (39S) subunit contains 16S rRNA and about 50 large subunit proteins (MRPLs). Disruption of their stability or assembly impairs mitochondrial translation and causes combined OXPHOS deficiency (COXPD) [1, 5, 8, 9, 10]. These mitochondrial ribosomopathies present with neurological symptoms and characteristic brain lesions in the basal ganglia, thalami, and brainstem. Other features include developmental delay, cardiomyopathy, lactic acidosis, and multi‐organ involvement [1, 5, 8]. Severity ranges from early death to survival into adulthood, depending on the genetic defect [1, 5, 11, 12, 13, 14, 15].

Combined Oxidative Phosphorylation Deficiency type 32 (COXPD32; MIM: 617664) is caused by pathogenic variants in *MRPS34* (MIM: 611994) [5, 11]. Dysfunction of *MRPS34* disrupts mitochondrial translation and results in energy deficiency [5]. As in other mitochondrial disorders, tissues with high energy demands, particularly the central nervous system, are most affected [16]. Located on chromosome 16p13.3, *MRPS34* encodes a component of the small (28S) subunit. Lake et al. identified four pathogenic *MRPS34* variants in six individuals with LS or LLS [5], all showing reduced *MRPS34* protein levels, destabilized mitoribosomes, and impaired mitochondrial translation. Expression of wild‐type *MRPS34* rescued the translation defect, confirming pathogenicity [5]. The clinical spectrum includes developmental delay, hypotonia, microcephaly, lactic acidosis, and neurodevelopmental regression [1, 5, 11, 14, 15]. While some individuals experience early death, others survive into adulthood, reflecting phenotypic variability [1, 5, 11, 15].

We report two new COXPD32 cases with presumed biallelic pathogenic autosomal recessive variants in *MRPS34* and provide an overview of clinical and genetic features associated with *MRPS34* variants.

## 2 Results

### 2.1 Case Presentations

The Danish individual, carrying two *MRPS34* variants, was submitted to GeneMatcher [17], which led to a match with an American individual; both are included in this study (Table S1).

#### 2.1.1 Clinical Features and Laboratory Findings

The first individual was female, born at 36–38 weeks' gestation, birth weight 1.96 kg, and second child of non‐consanguineous Danish parents. Pregnancy details, birth length, and head circumference were not reported; the neonatal period was unremarkable. At 5 months, irregular eye movements were noted; ophthalmologic evaluation showed pale optic discs and severely impaired vision. At 7 months, global developmental delay was observed. Brain magnetic resonance imaging (MRI), electroencephalogram, and hearing assessment were not performed. Breathing abnormalities were noted but not confirmed as apnea. No epileptic seizures were reported. Cardiac evaluation, including electrocardiography and echocardiography, was not reported. No disease‐specific treatment was reported. Urine analysis at 8 months revealed elevated alanine but no glucose, blood, protein, or ketones. At 9.5 months, she experienced an acute metabolic crisis and died within 3–4 h, likely due to ketoacidosis or hypoglycemia following infection. Plasma analysis of blood taken postmortem showed elevated amino acids, and urine analysis obtained postmortem showed hematuria, proteinuria, glucosuria, and lactic aciduria. No renal imaging or renal function assessment was reported during life. Autopsy revealed LS‐typical brain lesions affecting the midbrain, oculomotor nuclei, substantia nigra, pons, and olivary nuclei; neuroimaging was unavailable.

The second individual is a 10‐year‐old female, the first child of non‐consanguineous American parents; her ancestry is unknown. The pregnancy was complicated by bladder and chlamydia infections that were treated with antibiotics. She was delivered at 41 weeks via Cesarean for postdate induction with cardiac decelerations and oligohydramnios. Her birth weight was 3.5 kg, length 50.8 cm, and the neonatal period was unremarkable. Developmental delay was noted from infancy, including slow growth and abnormal eye movements from 3 months, which improved by 6–7 months. She later developed dysconjugate gaze, optic atrophy, and hypotonia. At age 10, cognitive and motor levels were equivalent to 15 and 9 months. She gained and subsequently lost motor milestones, including rolling, sitting, waving, and pulling to stand. Fine motor skills were limited, and feeding difficulties from 16 months required G‐tube placement at age 2, removed by 3.5 years. Neurologically, she had cerebellar and brainstem dysfunction, tremor, titubation, developmental regression, irregular breathing with breath‐holding, and mild obstructive sleep apnea. Her head circumference at 2 years was at the 2nd percentile; at 10 years, her weight and height were below the 3rd percentile. Additional findings included mild C‐shaped dextroscoliosis and joint hypermobility. MRI showed symmetric T2 hyperintensities in the medulla, pons, and midbrain, with mild medullary atrophy. Electroencephalogram showed intermittent frontotemporal rhythmic slow waves. No epileptic seizures were reported. Cardiac evaluation, including electrocardiography and echocardiography, was normal. Abdominal ultrasound at age 6 suggested mild nephrocalcinosis. Episodes of metabolic acidosis with low bicarbonate levels (7–8 mmol/L) were documented during illness or missed treatment, with partial improvement on bicarbonate therapy. Plasma lactate levels were variably elevated but were within the normal range on later assessments (1.3 mmol/L, reference < 2.2). Cerebrospinal fluid (CSF) analyses showed normal lactate and pyruvate levels, with isolated elevation of alanine. Glucosuria, hematuria, and hypoglycemia were not described. Quantitative renal function parameters were unavailable.

#### 2.1.2 Muscle Histology, Histochemistry, and Biochemistry

Respiratory chain (RC) enzyme analysis in skin fibroblasts of the first individual showed complex IV deficiency, indicating mitochondrial dysfunction; exact data for complex IV and the remaining complexes were unavailable. Adenosine triphosphate (ATP) synthesis in digitonin‐permeabilized fibroblasts, assessed using a previously described method [18], was markedly reduced compared to controls, with substrate‐specific residual activities of 5% with glutamate and malate, 5% with pyruvate and malate, and 4% with succinate in the presence of rotenone. Blue native polyacrylamide gel electrophoresis (BN‐PAGE) analyses demonstrated reduced assembly of complex I and markedly reduced assembly of complex IV compared to controls (results not shown).

In the second individual, muscle biopsy showed reduced oxidation of complexes III and IV in freshly isolated mitochondria, while adenine nucleotide translocator activity and ATP production were normal (values not reported). Histological stains for cytochrome c oxidase, succinate dehydrogenase, and nicotinamide adenine dinucleotide tetrazolium reductase revealed scattered fibers with focal subsarcolemmal mitochondrial accumulation. Plasma amino acids were normal except for elevated alanine and pyruvate.

#### 2.1.3 Molecular Genetic Studies

**FIGURE 1:**
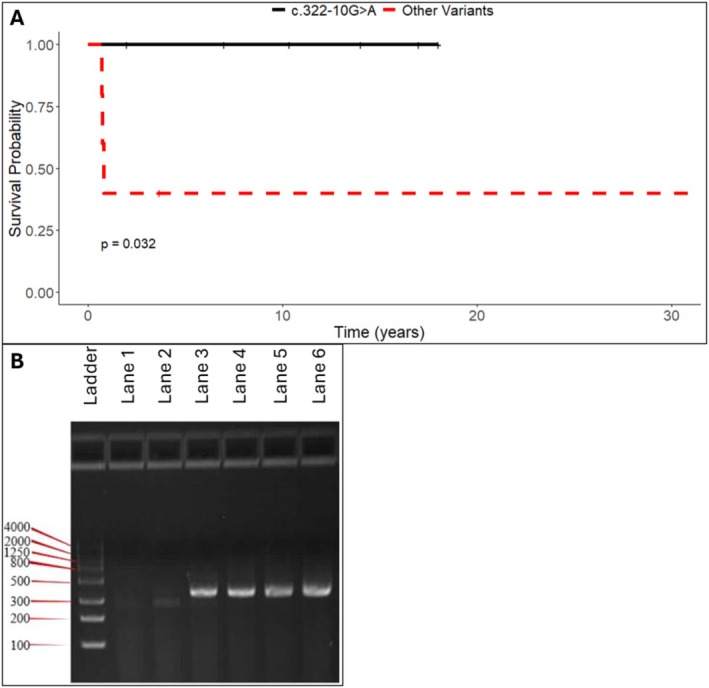
(A) Survival curve comparing c.322‐10G>A, p.(?) homozygotes (*n* = 6) with other *MRPS34* variants (*n* = 5), showing significantly longer survival in the c.322‐10G>A, p.(?) group. (B) PCR‐amplified *MRPS34* cDNA from individual 1 with (Lane 1) and without (Lane 2) NMD inhibitor, showing a very weak band of a smaller size compared to controls (Lanes 3–6).

In the first individual, mtDNA sequencing, chromosome analysis, and chromosomal microarray analysis (CMA) were unremarkable. Exome sequencing (ES) identified two *MRPS34* variants (GRCh38, NM_023936.2): c.94C>T, p.(Gln32*), a nonsense variant, previously reported [5], listed in dbSNP (rs763672163) [19], and observed heterozygously in 1789/1442020 alleles in gnomAD [20] v4.1.0 with no homozygotes; and c.364 + 2 T>C, p.(?), affecting the canonical donor splice site of intron 2 and predicted to disrupt splicing (SpliceAI donor loss score 0.75), reported in 4/1611864 alleles. Parental DNA was unavailable, but variants were presumed to be in trans based on phenotype and prior reports. RNA extracted from fibroblasts was reverse transcribed into cDNA, followed by PCR amplification of a fragment of *MRPS34* with an expected size of 374 bp. A severely decreased amount of shorter cDNA was found in the patient sample (Figure 1B), indicating that no normal transcript was produced and suggesting compound heterozygosity for the two *MRPS34* variants.

In the second individual, methylation analysis of 15q12 and CMA were normal. mtDNA sequencing revealed three likely benign homoplasmic variants, and initial nDNA gene testing, including a 101‐gene mitochondrial disease panel, showed no pathogenic variants. Genome sequencing using Genomic Unity identified a homozygous *MRPS34* splice‐region variant (c.322‐10G>A), p.(?), shown to abolish the intron 1 acceptor site and cause aberrant splicing [5]. This variant (rs563189672) occurs in 34/1604034 alleles in gnomAD [20], no homozygotes reported.

### 2.2 Variant Classification

All reported *MRPS34* variants were classified according to the American College of Medical Genetics and Genomics and Association for Molecular Pathology (ACMG/AMP) guidelines (Table 1). Most variants meet criteria for pathogenic classification, whereas one missense variant remains a variant of uncertain significance (VUS).

**TABLE 1:** Reported *MRPS34* variants in 11 individuals and their ACMG/AMP classifications.

Variant (HGVS)	ACMG criteria	Classification
c.37G>A, p.(Glu13Lys)	PS3_mod; PM2_sup; PM3_mod; PP4_mod; PP3_sup	Pathogenic
c.94C>T, p.(Gln32*)	PVS1_Vst; PS4_sup; PP4_mod	Pathogenic
c.223G>C, p.(Gly75Arg)	PM2_sup; PP3_sup; PP4_mod	VUS
c.321 + 1G>T, p.(Val100_Gln107del)	PVS1_Vst; PM2_sup; PP4_mod	Pathogenic
c.322‐10G>A, p.(Asn108Leufs*12); p.(Asn108Glyfs*50)	PVS1_Vst; PS4_sup; PM2_sup; PP4_mod	Pathogenic
c.364 + 2 T>C, p.(?)	PVS1_Vst; PM2_sup; PP4_mod	Pathogenic
c.580C>T, p.(Gln194*)	PVS1_Vst; PM2_sup; PM3_mod; PP4_mod	Pathogenic

*Note:* Criteria were applied according to ACMG/AMP guidelines [21].

Abbreviations: ACMG/AMP = American College of Medical Genetics and Genomics/Association for molecular pathology; mod = moderate; sup = supporting; VUS = variant of uncertain significance; Vst = very strong.

## 3 Clinical Spectrum and Affected Systems

Including this report, 11 individuals with *MRPS34*‐related disease have been reported (Table 1, Table 2, Table S1).

**TABLE 2:** Clinical findings in 11 individuals with *MRPS34* variants.

	Clinical parameter	Data overall (*n* = 11)
Perinatal/demographics	Sex	Female: 7/11 (63.6%); Male: 4/11 (36.4%)
	Age at presentation	10/10 (100%); median 6 months (10 days–1 year 9 months)
	Age at last evaluation	11/11 (100%); median 7 years (4 months–34 years)
	Deceased (age)	3/11 (27.3%); median 9 months (8.5 months–9.5 months)
Neurodevelopment	Motor delay	11/11 (100%)
	Intellectual delay	7/7 (100%)
	Language delay/disorder	8/8 (100%)
	Developmental regression	7/8 (87.5%); median 1 year 4 months (4 months–5 years)
	Movement disorder	6/9 (66.7%)
	Abnormal reflexes	5/5 (100%)
	Hypotonia	6/6 (100%)
	Microcephaly	5/5 (100%)
Neuroimaging/central nervous system	Abnormal magnetic Resonance imaging	10/10 (100%)
	Basal ganglia lesions	3/10 (30.0%)
	Thalami lesions	4/10 (40.0%)
	Brainstem lesions	10/11 (90.9%)
	Cerebellar lesions	2/10 (20.0%)
	Brain atrophy	3/4 (75.0%)
Ophthalmologic	Strabismus	6/6 (100%)
	Optic atrophy	3/7 (42.9%)
Feeding/GI	Feeding/GI symptoms	8/9 (88.9%)
	G‐tube placement	4/9 (44.4%); median 3.5 years (1 year 11 months–12 years)
Metabolic/laboratory workup	Metabolic crisis	5/6 (83.3%)
	Lactic acidosis	10/11 (90.9%)
	Decreased respiratory chain enzyme activity	7/7 (100%)
	Abnormal cerebrospinal fluid	4/4 (100%)
Other	Abnormal breathing (awake)	4/9 (44.4%)
	Renal abnormalities	3/3 (100%)
	Dysmorphic features	5/6 (83.3%)
	Scoliosis	4/4 (100%)
	Sleep apnea (mild/suspected)	3/4 (75.0%)

*Note:* Clinical data were incomplete for some features; available patient numbers are indicated (*n* < 11). Proportions are based on individuals with available data only; features not included were either not reported or not assessed.

Abbreviations: D = days; G‐tube = gastrostomy tube; GI = gastrointestinal; m = months; y = years.

Disease onset was early (median 6 months, *n* = 10), and three individuals (27.3%) died at a median age of 9 months. All 11 individuals showed delayed motor development. Developmental regression occurred in 87.5% (7/8) typically before 1.5 years, and among those assessed, all had intellectual disability (7/7) and language delay/disorder (8/8). Common neurological features included hypotonia (6/6), abnormal reflexes (5/5), microcephaly (5/5), movement disorders (6/9), and tremor (5/9).

Neuroimaging or autopsy revealed brain abnormalities in all 11 individuals, primarily in the brainstem (11/11; 100%). Thalamic lesions (4/10), basal ganglia lesions (3/10), cerebellar lesions (2/10), and brain atrophy (3/4) were also observed. Ophthalmological involvement included strabismus (6/6), optic atrophy (3/7), vision loss (3/3), ptosis (2/3), and nystagmus (1/2).

Gastrointestinal and feeding issues were common (8/9 and 7/8), with a gastrostomy tube required in 4/9 at median age 3.5 years. Additional features included renal abnormalities (3/3), dysmorphic features (5/6), scoliosis (4/4), joint contractures (3/4), sleep apnea, hypogonadotropic hypogonadism, and adrenal insufficiency. Metabolic abnormalities included lactic acidosis (10/11), metabolic crises (5/6), and decreased RC enzyme activity (7/7). CSF abnormalities were reported in all tested (4/4).

Among reported *MRPS34* variants, c.322‐10G>A, p.(?) is associated with a distinct disease course. Six out of 11 individuals are homozygous for this variant, and despite the small sample size, Kaplan–Meier analysis showed significantly prolonged survival in this group compared with other *MRPS34* genotypes (Figure 1A; *p* < 0.05). Homozygosity for c.322‐10G>A, p.(?) is consistently associated with severe neurodevelopmental impairment, indicating slower progression rather than a mild phenotype.

## 4 Discussion

This study describes two additional individuals with presumed biallelic *MRPS34* variants associated with LS and RC deficiency, expanding the number of reported cases to 11 (Table 2, Table S1).

Across 11 individuals, *MRPS34*‐related disease is a multisystem disorder with universal developmental delay, with language delay, hypotonia, movement disorders, and brainstem lesions, while cardiac and adrenal involvement appear less frequent. MRI abnormalities in the brainstem reinforce *MRPS34*'s role in LSS. Lactic acidosis and RC deficiencies were common, while features such as optic atrophy and epilepsy may reflect variable expressivity or incomplete documentation. Incomplete clinical data may obscure true prevalence, but the pattern supports a neurometabolic disorder with brainstem‐predominant imaging, developmental regression, and mitochondrial dysfunction.

Among individuals with available RC data, complex IV deficiency is the most consistent abnormality, often accompanied by reduced activity of complexes I and/or III. In Individual 1, BN‐PAGE demonstrated reduced assembly of complex I and markedly reduced assembly of complex IV compared to controls. Previous studies of *MRPS34* deficiency [5] showed reduced OXPHOS complex assembly on BN‐PAGE and delayed formation of complex IV on pulse‐chase analysis, consistent with the abnormalities observed here.

Individuals homozygous for c.322‐10G>A, p.(?) show a developmental spectrum, ranging from early regression with milestone loss to more stable courses without regression [1, 5, 11, 14, 15]. While long‐term survival is possible [1, 14, 15], data indicate persistent neurodevelopmental impairment.

Functionally, c.322‐10G>A, p.(?) creates a novel acceptor site at position c.322–9 and has been shown to result in aberrant splicing with residual wild‐type transcript, consistent with a hypomorphic effect [5]. Residual *MRPS34* expression likely contributes to the survival advantage. The variant is overrepresented among reported COXPD32 cases relative to its low allele frequency in population databases [20], and its frequency, including in individuals from Puerto Rico [5], is compatible with a founder effect as suggested [5], although this cannot be confirmed with current data.

Mitochondrial ribosomal disorders represent a subgroup of mitochondrial disease caused by impaired protein synthesis due to defects in mitoribosomal proteins and are typically associated with early‐onset neurodevelopmental disease and combined OXPHOS deficiencies [5, 8]. This is consistent with mammalian mitoribosome assembly studies showing that several disease‐associated mitoribosomal proteins are incorporated at early stages of assembly, suggesting a link between early assembly defects and severe clinical presentation [22]. *MRPS34*‐related disease fits within this subgroup due to its primary defect in mitochondrial translation and is frequently characterized by brainstem involvement, complex IV deficiency, and phenotypic variability [1, 5, 11, 14, 15].

The clinical variability, from early mortality to prolonged survival in hypomorphic variants, supports a genotype‐dependent disease course. Management remains supportive, as no disease‐specific therapies are available. Accurate genetic diagnosis remains essential for clinical management, prognosis, and counseling, and further functional studies may clarify the disease.

## Author Contributions

Alberte Aspaas Lundquist: conceptualization, methodology, data collection and structuring, formal analysis, original draft writing. Elsebet Østergaard: conceptualization, methodology, data collection and structuring, supervision, resources, original draft writing. Sumit Parikh: patient data, manuscript review and editing. Flemming Wibrand and Thomas van Overeem Hansen: formal analysis, data interpretation, manuscript review and editing.

## Funding

The authors have nothing to report.

## Ethics Statement

The Danish family was lost to follow‐up, but previously gave oral consent. Patient consent for the American family was not required in accordance with the policies of the local Institutional Review Board for deidentified case reports.

## Conflicts of Interest

The authors declare no conflicts of interest.

## Supporting information


**Table S1:** Clinical and biochemical data of all 11 individuals reported to date with *MRPS34*‐related disease. Proportions are based on individuals with available data only; features not included were either not reported or not assessed.

## Data Availability

The data that support the findings of this study are available in the Supporting Information of this article, and additional data are available from the corresponding author upon reasonable request.
